# Nomogram for predicting cognitive impairment in middle-aged and elderly individuals with self-reported hearing loss: Insights from the longitudinal CHARLS cohort

**DOI:** 10.1016/j.bjorl.2025.101751

**Published:** 2026-01-07

**Authors:** Cheng Li, Yan Mei, Wei Li, Dan Liu

**Affiliations:** a Ezhou Central Hospital, Department of Otolaryngology, Ezhou, China; b Huangshi Central Hospital (Affiliated Hospital of Hubei Polytechnic University), Department of Otolaryngology, Huangshi, China

**Keywords:** Cognitive impairment, Hearing loss, Middle-aged and older adults, Nomogram, CHARLS

## Abstract

**Objective:**

To develop and evaluate a predictive model for cognitive impairment, assessed by a brief cognitive test, among middle-aged and older adults with self-reported hearing loss, aiming to facilitate early identification and targeted intervention in high-risk populations.

**Methods:**

This study utilized data from the 2011–2018 waves of the China Health and Retirement Longitudinal Study, including 1,093 individuals aged 45 and above with self-reported hearing impairment but normal cognition at baseline. The average follow-up duration was 5.2-years. Cognitive impairment was defined as a total cognitive score at least one standard deviation below the mean for the corresponding age group. Univariate and multivariate Cox proportional hazards models were used to identify independent predictors, and a nomogram was constructed based on significant variables. Model performance was assessed using calibration curves, time-dependent Concordance indices (C-index), and the Area Under the receiver operating Characteristic Curve (AUC). Risk stratification analysis was conducted across various sociodemographic subgroups.

**Results:**

During follow-up, 152 individuals (13.9%) developed cognitive impairment. Higher education level, urban residence, and participation in leisure social activities (e.g., playing mahjong) were independently associated with lower risk of cognitive impairment. The nomogram demonstrated good discriminative performance, with AUCs of 0.728, 0.762, and 0.768, and corresponding time-dependent C-indices of 0.719, 0.761, and 0.767 at 3-, 5-, and 6-year follow-up points, respectively. Calibration plots indicated good agreement between predicted and observed risks. Risk scores varied significantly across subgroups defined by sex, age, education level, and residential location. Kaplan–Meier analyses confirmed the model’s effective risk stratification capability.

**Conclusion:**

This study presents the first predictive model tailored to cognitive risk heterogeneity among older adults with self-reported hearing loss. By incorporating a brief cognitive test, the nomogram demonstrated reliable performance and holds potential for use in risk stratification, population screening, and personalized preventive interventions.

**Level of evidence:**

2 ‒ Prospective cohort study based on the longitudinal CHARLS data.

## Highlights

• A nomogram predicts cognitive decline in self-reported hearing-impaired older adults.
• Education, urban living, and social activity are key protective factors.
• The model shows good performance (AUC 0.728–0.768; C-index >0.71).
• Risk scores differ significantly across demographic subgroups.
• The nomogram facilitates early identification and targeted prevention.

## Introduction

Cognitive impairment, as a precursor to dementia, has become a major public health issue threatening the health of middle-aged and elderly populations.^1^ Statistics show that the prevalence of moderate cognitive loss is approximately 6% among individuals aged 60–64, and rises to about 25% among those aged 80‒84.^2^ Related studies indicate that in 2015, the economic cost of cognitive impairment in China amounted to $18 billion, imposing a significant economic burden on individuals, families, and society.^3^ The research also points out that a 10%–25% reduction in risk factors, including those related to cognitive impairment, could potentially prevent up to 1.1–3 million cases of Alzheimer’s disease worldwide.^4^ Therefore, identifying the factors that influence cognitive impairment in middle-aged and elderly individuals and making effective predictions is of significant clinical importance.

Recent studies have found that hearing impairment has been confirmed as an independent risk factor for cognitive decline and the onset of dementia.^5^, ^6^, ^7^ A multicenter, randomized controlled trial indicated that hearing intervention might help reduce cognitive changes over 3-years in older adults at high risk for cognitive decline.^8^ Individuals with hearing loss frequently experience social isolation, depression, and reduced participation, which can increase cognitive load and decrease stimulation, thereby accelerating cognitive deterioration and dementia onset.^9^, ^10^, ^11^ The mechanisms linking hearing loss to cognitive decline are multifaceted. Specifically, sensorineural hearing loss, which accounts for approximately 90% of cases, is the subtype most strongly associated with cognitive impairment, potentially through mechanisms such as increased cognitive load, comorbidities, and sensory deprivation.^12^^,^^13^ However, current research primarily focuses on the overall relationship between hearing loss and cognitive impairment, with only limited evidence exploring factors that may further elevate risk in this population. For example, one study reported that middle-aged individuals with hearing loss who also had cardiovascular disease exhibited a significantly increased risk of dementia.^14^ Overall, predictive models of cognitive impairment specifically targeting individuals with hearing loss remain scarce. Identifying early predictive factors in this high-risk group and developing effective predictive tools holds therefore significant clinical value.

Currently, there is a lack of research on the prediction of cognitive impairment risk in hearing-impaired middle-aged and elderly populations, which somewhat limits early identification and precise intervention for this group. The nomogram, as a visual risk prediction tool, integrates multiple clinical characteristics and provides individuals with a quantified risk score, offering strong potential for personalized prediction and clinical application.^15^ Based on this, our study employed a prospective design and selected individuals from the China Health and Retirement Longitudinal Study (CHARLS), using data from four waves of follow-up between 2011 and 2018. Through Cox regression analysis, we developed a nomogram model to predict the risk of cognitive impairment in self-reported hearing-impaired middle-aged and elderly individuals. This model aims to provide scientific evidence for the early identification of cognitive impairment in self-reported hearing-impaired populations and to support the development of clinical intervention strategies.

## Methods

### Data cleaning and sample selection process

**Fig. 1:**
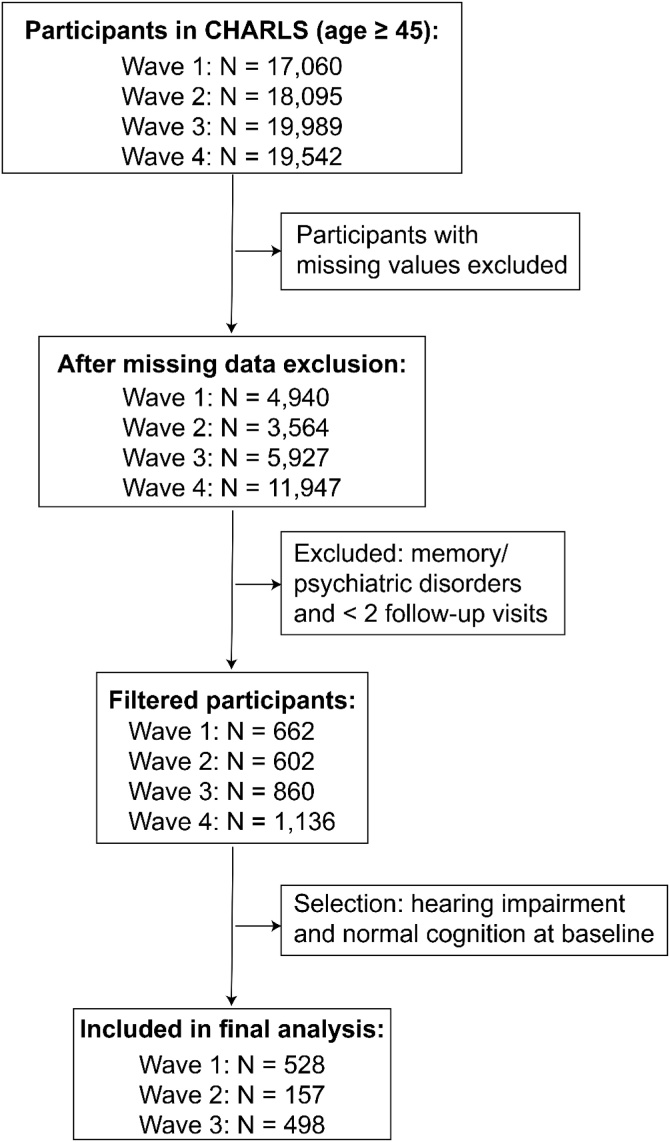
Data cleaning flowchart.

This study utilized data from the China Health and Retirement Longitudinal Study (CHARLS), a nationally representative cohort of individuals aged 45-years and older. Participants aged 45–59 years were classified as middle-aged, and those aged 60-years and above were classified as elderly. In this study, the final analytic sample included participants aged 45 to 87-years. Data were drawn from four survey waves of CHARLS, with the baseline survey conducted in 2011 (Wave 1) and follow-ups in 2013 (Wave 2), 2015 (Wave 3), and 2018 (Wave 4), at intervals of approximately 2–3 years. Variables included sociodemographic characteristics (age, gender, education level, marital status, and residence), lifestyle factors (current smoking, current drinking, sleep duration, and Total Metabolic Equivalent [METs]), social participation (eight types of social activities), psychological well-being (depressive symptoms, impairment in Instrumental Activities of Daily Living [IADL], and hope for the future), and health status (hypertension, diabetes, stroke, and dyslipidemia). Detailed definitions and classifications of these variables are presented in Table 1. After excluding participants with missing values or fewer than two follow-up waves, 17,001 individuals remained. Among them, 3,179 participants with self-reported hearing impairment at baseline were identified. After further excluding those with cognitive impairment at baseline, a final cohort of 2,676 hearing-impaired individuals with normal cognition was established. Survival data were constructed to track the first onset of cognitive impairment during follow-up. Ultimately, 1,093 individuals with complete outcome data were included in the final analysis. The detailed sample selection process is illustrated in Fig. 1. The methodology and data collection procedures of CHARLS have been described in detail in a prior publication.^16^ Ethical approval was obtained from the Ethical Review Committee of Peking University (IRB00001052–11015), and all participants provided written informed consent.

**Table 1:** Baseline characteristics of hearing-impaired participants with and without cognitive impairment.

Characteristics	Without cognitive impairment (n = 941)	With cognitive impairment (n = 152)	p-value
Age, years	55.83 (8.39)	56.59 (8.10)	0.299
Gender, n (%)			0.002
Female	403 (42.8)	86 (56.6)	
Male	538 (57.2)	66 (43.4)	
Education level, n (%)			<0.001
Below primary	159 (16.9)	67 (44.1)	
Primary	215 (22.8)	43 (28.3)	
Middle school	310 (32.9)	33 (21.7)	
High school or above	257 (27.3)	9 (5.9)	
Marital status, n (%)			0.049
Other	65 (6.9)	18 (11.8)	
Married	876 (93.1)	134 (88.2)	
Residence, n (%)			<0.001
Urban	482 (51.2)	51 (33.6)	
Rural	459 (48.8)	101 (66.4)	
Current drinking, n (%)	378 (40.2)	54 (35.5)	0.319
Current smoking, n (%)	321 (34.1)	44 (28.9)	0.246
Social activity 1, n (%)	432 (45.9)	62 (40.8)	0.276
Social activity 2, n (%)	289 (30.7)	28 (18.4)	0.003
Social activity 3, n (%)	161 (17.1)	20 (13.2)	0.272
Social activity 4, n (%)	132 (14.0)	14 (9.2)	0.136
Social activity 5, n (%)	48 (5.1)	2 (1.3)	0.062
Social activity 6, n (%)	63 (6.7)	2 (1.3)	0.016
Social activity 7, n (%)	14 (1.5)	1 (0.7)	0.66
Social activity 8, n (%)	11 (1.2)	1 (0.7)	0.887
IADL impairment, n (%)	56 (6.0)	16 (10.5)	0.053
Hope for the future, high (n, %)	536 (57.0)	72 (47.4)	0.034
Depressive symptoms (CESD ≥ 10), n (%)	141 (15.0)	34 (22.4)	0.029
Sleep duration, hours	6.63 (1.58)	6.18 (1.99)	0.002
Hypertension, n (%)	206 (21.9)	33 (21.7)	1.000
Diabetes, n (%)	62 (6.6)	7 (4.6)	0.451
Stroke, n (%)	12 (1.3)	3 (2.0)	0.756
Dyslipidemia, n (%)	125 (13.3)	17 (11.2)	0.559
Total METs quartiles, n (%)			0.001
Q1	233 (24.8)	41 (27.0)	
Q2	254 (27.0)	19 (12.5)	
Q3	223 (23.7)	50 (32.9)	
Q4	231 (24.5)	42 (27.6)	

IADL, Instrumental Activities of Daily Living; CESD, Center for Epidemiologic Studies Depression Scale; MET, Metabolic Equivalent Task level.

### Variable definition and measurement

This study extracted 24 variables from the CHARLS dataset (Table 1). Details of social activity variables are provided in Supplementary Table 1.Cognitive function was assessed using a simplified cognitive test derived from the Mini-Mental State Examination (MMSE, which has been widely used in CHARLS-based studies.^20^^,^^21^ The assessment comprised three domains: orientation and attention [Telephone Interview of Cognitive Status-10 (TICS-10), 10-points], episodic memory (word recall, 10-points), and visuospatial ability (figure drawing, 1-point), with a total score ranging from 0 to 21.^17^, ^18^, ^19^ However, it should be noted that this scale is less sensitive for detecting mild cognitive decline, which should be considered when interpreting our findings. Cognitive impairment was defined as a score below one standard deviation from the age-specific group mean.^22^^,^^23^ Hearing status was self-reported using the question: “How is your hearing? (If you frequently wear a hearing aid, how is your hearing while wearing it? If you do not frequently wear a hearing aid, how is your hearing without it?) Is it excellent, good, fair, or poor?” Participants answering “fair” or “poor” were classified as having hearing impairment. Physical activity level was categorized into quartiles (Q1–Q4) based on total METs. IADL were assessed across five domains; any impairment was coded as 1 (binary). Depressive symptoms were evaluated using the CES-D 10-item scale, with a score ≥10 indicating depressions.^24^ Life hope was coded as a binary variable (scores 4–5 = high hope; 1–3 = low hope). All variables underwent consistency checks and standardization prior to modeling.

### Statistical analysis

All statistical analyses were performed using R version 4.3.0, with key packages including survival (v3.5–7), rms (v6.7−0), pec (v3.4.4), timeROC (v0.4), survminer (v0.4.9), and ggplot2 (v3.4.4). Baseline characteristics were compared by cognitive status. Continuous variables were analyzed using independent *t*-tests or Mann–Whitney *U* tests, and ANOVA or Kruskal–Wallis tests for ≥3 groups, based on distribution. Categorical variables were compared using Chi-Square tests. Univariate Cox regression was used to screen variables associated with cognitive impairment (p < 0.05). Significant variables were entered into a multivariate Cox model to identify independent predictors. A nomogram was constructed based on the multivariate results, and individual risk scores were calculated. Model performance was evaluated via: 1) Calibration curves at 3-, 5-, and 6-years (rms); 2) Time-dependent C-index (pec); and 3) ROC curves and AUCs with 95% CIs (timeROC). Individuals were stratified into high- and low-risk groups by median score. Kaplan-Meier curves were plotted by subgroup to assess stratification, and log-rank tests were used for group comparisons. All statistical tests were two-sided, with p < 0.05 considered significant.

## Results

### Baseline profiles by cognitive function in individuals with self-reported hearing loss

Among the 1,093 participants with self-reported hearing impairment included in the analysis, 152 (13.9%) developed cognitive impairment during the follow-up period. We compared the baseline characteristics between participants with normal cognition (n = 941) and those with cognitive impairment (n = 152), as shown in Table 1. There was no significant difference in mean age between the two groups (55.83 vs. 56.59 years, p = 0.299). However, gender distribution differed significantly, with a higher proportion of females in the cognitively impaired group (56.6% vs. 42.8%, p = 0.002). Education level was strongly associated with cognitive status: individuals with cognitive impairment were more likely to have a lower education level (primary school or below: 72.4% vs. 39.7%, p < 0.001). Marital status also differed, with a higher proportion of married individuals in the cognitively normal group (93.1% vs. 88.2%, p = 0.049). Furthermore, a significantly greater proportion of participants with cognitive impairment lived in rural areas (66.4% vs. 48.8%, p < 0.001). Regarding lifestyle factors, current smoking and drinking did not differ significantly between groups (p > 0.05). Sleep duration was slightly lower in the cognitively impaired group (6.18 vs. 6.63 h, p = 0.002). In terms of psychosocial factors, participants with cognitive impairment had a lower level of hope for the future (47.4% vs. 57.0%, p = 0.034) and a higher prevalence of depressive symptoms (CESD-10 ≥ 10: 22.4% vs. 15.0%, p = 0.029). Participation in social activities was generally lower in the cognitively impaired group. Notably, participation in leisure activities such as Mahjong, cards, chess, or attending a community room was significantly lower in the cognitively impaired group (18.4% vs. 30.7%, p = 0.003). Engagement in volunteering or charitable activities also showed a significant difference (1.3% vs. 6.7%, p = 0.016). Other social activities, including visiting friends, outdoor exercise, and informal help to others, were slightly more common in the cognitively normal group, although the differences were not statistically significant. With respect to health-related variables, impairment in IADL was more prevalent in the cognitively impaired group (10.5% vs. 6.0%, p = 0.053). No significant group differences were observed for chronic conditions including hypertension, diabetes, stroke, or dyslipidemia (p > 0.05). Lastly, when analyzing physical activity levels based on Metabolic Equivalent (MET) quartiles, the cognitively impaired group had a lower proportion of participants in the second quartile (moderate activity, 12.5% vs. 27.0%, p = 0.001). Although a higher proportion of cognitively impaired participants were classified in the upper quartiles (Q3 and Q4), the differences were not statistically significant. In summary, participants who developed cognitive impairment were more likely to have lower educational attainment, reside in rural areas, report lower psychological well-being, exhibit depressive symptoms, and engage less frequently in social activities. These factors may play an important role in cognitive decline among individuals with hearing loss.

### Risk factors for cognitive dysfunction in hearing-impaired elderly individuals

**Fig. 2:**
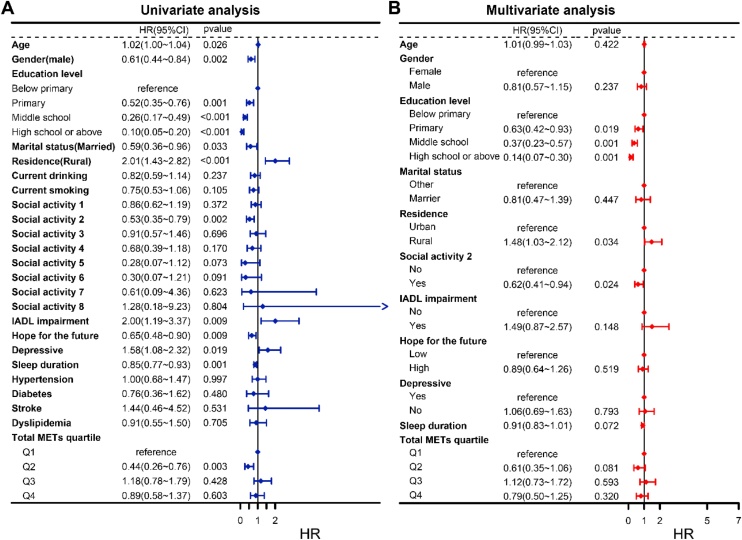
Forest plot of factors influencing cognitive impairment risk in older adults with hearing loss. (A) Univariate Cox regression; (B) Multivariate Cox regression.

We first performed univariate Cox regression analysis to identify potential risk factors associated with the occurrence of cognitive dysfunction in self-reported hearing-impaired individuals (Fig. 2A). The results showed significant associations between multiple variables and the occurrence of cognitive dysfunction, as assessed by a simple cognitive screening tool. Compared to individuals with education levels below elementary school, those with higher education (elementary, secondary, and high school or above) had a significantly lower risk of cognitive dysfunction (HR = 0.52, 0.26, and 0.10, all p < 0.001). The risk of cognitive dysfunction was significantly higher in women compared to men (HR = 0.61, p = 0.002). Rural residents had a significantly higher risk of cognitive dysfunction than urban residents (HR = 2.01, p < 0.001). Additionally, being married (HR = 0.59, p = 0.033), depressive symptoms (HR = 1.58, p = 0.018), lower future hope (HR = 0.65, p = 0.009), IADL impairment (HR = 2.00, p = 0.009), and shorter sleep duration (HR = 0.85, p = 0.0007) were also significantly associated with an increased risk of cognitive dysfunction. In terms of social participation, involvement in community leisure activities (e.g., playing Mahjong, chess) was significantly associated with a lower risk of cognitive dysfunction (HR = 0.53, p = 0.002). Moderate physical activity (Q2 vs. Q1) also had a protective effect (HR = 0.44, p = 0.003). After incorporating these statistically significant variables into a multivariate Cox regression model (Fig. 2B), some factors still showed independent predictive value. Higher education levels continued to significantly reduce the risk of cognitive dysfunction (HR = 0.63, 0.37, and 0.14, all p < 0.05), and rural residence remained associated with a higher risk (HR = 1.48, p = 0.034). Furthermore, participation in social leisure activities (e.g., Mahjong, chess) remained associated with a lower risk of cognitive dysfunction in the multivariate model (HR = 0.62, p = 0.024). Other variables, such as marital status, depressive symptoms, IADL impairment, hope, sleep duration, and physical activity levels, showed trends towards association with cognitive status but did not reach statistical significance (p > 0.05). In summary, higher education, urban residence, and participation in community leisure activities (e.g., Mahjong, chess) may be important protective factors against cognitive dysfunction in self-reported hearing-impaired individuals. This suggests that improving education, living environments, and promoting social participation could delay or reduce the risk of cognitive dysfunction in this population.

### Evaluation of the nomogram model's predictive performance

**Fig. 3:**
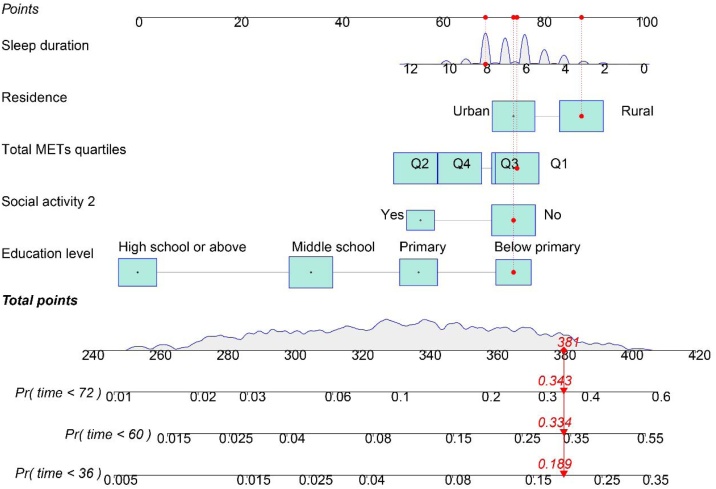
Nomogram model for predicting cognitive impairment risk in older adults with hearing loss.

**Fig. 4:**
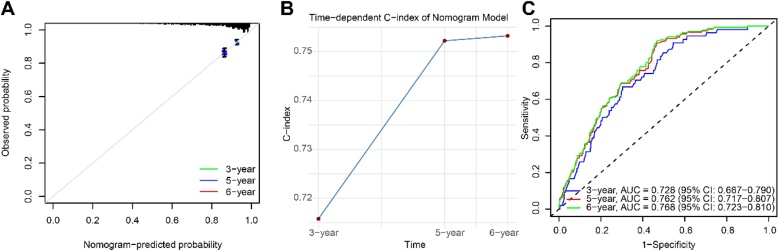
Evaluation of the predictive performance of the nomogram model. (A) Calibration curves showing good agreement between the predicted and actual probabilities at 3-year, 5-year, and 6-year follow-up points; (B) Time-dependent C-index curves reflecting the model's good discrimination ability at 3-year, 5-year, and 6-year follow-up points; (C) ROC curves and corresponding AUC values at different time points, further validating the model's high predictive efficiency and stability.

We integrated statistically significant variables from the multivariate regression analysis (education level, residence, sleep duration, physical activity level, and participation in leisure social activities) to construct a nomogram model for predicting the risk of cognitive dysfunction in self-reported hearing-impaired elderly individuals (Fig. 3). The model demonstrated good predictive accuracy and discrimination at 3-, 5-, and 6-years. Calibration curves showed that the predicted probabilities closely matched the actual event probabilities, indicating good model fit (Fig. 4A). Time-dependent C-index analysis further confirmed the model’s discriminatory ability, with C-index values of 0.719, 0.761, and 0.767 at the 3-year, 5-year, and 6-year time points, respectively, all indicating relatively high performance (Fig. 4B). Additionally, ROC curve analysis revealed that the model's AUC at these three time points was 0.728 (95% CI 0.667–0.790), 0.762 (95% CI 0.717–0.807), and 0.768 (95% CI 0.723–0.810), suggesting that the model maintains stable and robust predictive performance across different follow-up time points (Fig. 4C). In summary, the nomogram model developed in this study demonstrates high accuracy and clinical potential in predicting the risk of cognitive dysfunction in self-reported hearing-impaired elderly individuals.

### Distribution of nomogram model scores in different population characteristics and its survival prediction efficacy

**Fig. 5:**
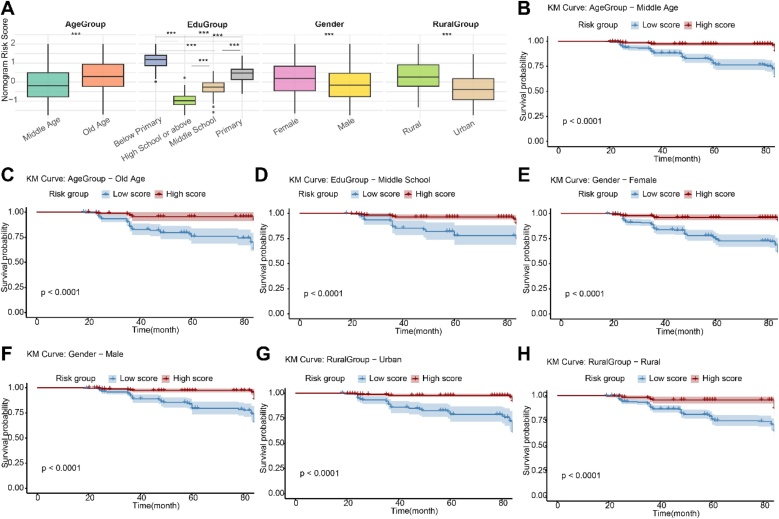
Distribution of nomogram scores in different population subgroups and its survival prediction efficiency. (A) Distribution of nomogram scores across different population characteristics (sex, age, education level, and residential location). (B—H) Kaplan-Meier survival curves showing significant differences in cognitive impairment risk between high-risk and low-risk groups stratified by scores in each subgroup.

There were significant differences in the distribution of nomogram model risk scores across different sociodemographic subgroups. We compared the score differences across four categorical variables: gender, age group, education level, and residence. The results showed that all comparisons were statistically significant (all p < 0.05), indicating that the model's risk score effectively distinguishes the risk levels among different population characteristics (Fig. 5A). Furthermore, we divided the population into high-risk and low-risk groups based on the median model score and performed risk stratification analysis by plotting Kaplan-Meier survival curves within each subgroup. The results revealed that, except for the comparison between the “elementary school” and “high school and above” groups, which did not reach statistical significance, high-risk groups in all other education, gender, age, and residence subgroups experienced cognitive dysfunction significantly earlier during the follow-up than the low-risk groups (Fig. 5B‒H, p < 0.05). It should be noted that, since all individuals in the “below elementary school” group were classified into the high-risk group, there was no low-risk control group for comparison, and thus, no Kaplan-Meier curve comparison was conducted for this group. These results suggest that the nomogram model not only exhibits distinct distributions in different sociodemographic subgroups but also demonstrates good survival prediction efficacy and risk stratification ability.

## Discussion

This study, based on nationally representative data from the CHARLS and using simplified assessment tools, is the first to provide a preliminary systematic assessment of the risk factors for cognitive impairment in middle-aged and older adults with self-reported hearing loss and to construct a nomogram for individualized risk prediction. We found that approximately 13.9% of individuals with self-reported hearing loss developed cognitive impairment over an average follow-up of 5-years, indicating that this population is not homogeneous but rather exhibits significant heterogeneity. Previous cross-sectional studies have explored cognitive impairment risk,^25^, ^26^, ^27^ but by utilizing longitudinal data, our study fills the gap left by prior research in identifying cognitive risk disparities within the hearing-impaired group. This highlights the importance of conducting individualized risk assessments within this specific population.

Univariate Cox regression analysis identified several factors significantly associated with cognitive impairment risk, including age, sex, education, marital status, residential location, sleep duration, IADL limitations, hope, depressive symptoms, physical activity, and participation in leisure social activities. Educational attainment, a well-established protective factor against cognitive decline, remained a strong predictor even after adjusting for other variables.^28^^,^^29^ This effect is likely due to the role of education in enhancing cognitive reserve, promoting neuroplasticity, improving health literacy, and possibly modulating the gut microbiota.^30^^,^^31^ Urban residents had a significantly lower risk of cognitive impairment compared to those living in rural areas, highlighting the critical impact of the environment on cognitive aging. A previous study has also shown higher rates of mild cognitive impairment and dementia in rural populations, especially among women.^32^ This discrepancy may be due to factors such as limited healthcare access, weaker social support, and a lack of cognitive stimulation in rural areas.^33^^,^^34^ Interestingly, participation in structured leisure activities such as playing mahjong or chess remained an independent protective factor in the multivariate model. These activities combine cognitive engagement, emotional expression, and social interaction, and likely enhance cognitive resilience through multiple mechanisms. This finding aligns with the “cognitive-social interaction model”, suggesting that social engagement not only regulates emotions but also stimulates cognitive processes and slows cognitive decline.^35^ Prior research also highlights a positive feedback loop between cognitive activity and brain health, where higher cognitive function encourages continued engagement in intellectually stimulating tasks.^36^ However, variables like marital status, female sex, sleep duration, hope, IADL limitations, physical activity, and depressive symptoms, though significant in univariate analyses, lost statistical significance in the multivariate model. This may indicate that their effects are mediated by education, residential location, or social participation, or that multicollinearity and interaction effects reduced their independent contribution. Depressive symptoms and IADL limitations are often seen as early signs or comorbidities of cognitive impairment, rather than independent risk factors, which aligns with our results.^37^^,^^38^ Although sleep duration and sense of hope did not reach statistical significance in the multivariate model, their directional association with cognitive impairment was consistent with prior studies, suggesting they may still play a role.^39^^,^^40^ Future research with larger samples and more precise measurements could provide further insight into their effects. This study emphasizes the importance of addressing the unique mechanisms of cognitive decline in hearing-impaired individuals. While common risk factors for cognitive impairment in the elderly are relevant, they may be less predictive in individuals with hearing loss. For these individuals, cognitive decline may be more influenced by sensory deprivation, communication barriers, and social isolation. Tailored predictive models, like the one developed in this study, are essential for meeting the specific needs of this high-risk population.

Despite the strengths of this study, including its nationally representative sample and robust internal validation, several limitations must be acknowledged. First, hearing loss was assessed by self-report rather than objective audiometric testing, which may introduce misclassification bias and prevents characterization of hearing loss severity or type. Second, cognitive function was evaluated using a simplified 21-point scale adapted from the MMSE, which is widely used in CHARLS-based studies but may lack sensitivity in detecting mild or early-stage cognitive decline. Third, variables such as depression, social participation, and lifestyle factors were self-reported, introducing the potential for recall or reporting bias. Fourth, external validation has not yet been conducted, and the model’s generalizability requires confirmation in independent cohorts.

## Conclusion

This study developed and validated the first predictive model for cognitive impairment in middle-aged and older adults with self-reported hearing loss. The model showed good discriminative ability and identified key clinical risk factors, supporting its potential use in early detection and targeted prevention in community settings. Nevertheless, as both hearing loss and cognitive outcomes were assessed using simplified tools, future studies incorporating objective audiological evaluations, comprehensive neurocognitive batteries, biomarkers, and external validation are warranted to improve accuracy and generalizability.

## ORCID ID

Li Cheng: 0009-0000-8584-3223

Yan Mei: 0009-0008-3735-145X

Wei Li: 0009-0002-0553-3087

## Funding

This work was supported by the Hubei Provincial Natural Science Foundation of China (grant nº 2022CFB498).

## Data availability statement

We declare that all data are available in repository.

## Declaration of competing interest

The authors declare no conflicts of interest.
