# Supplementary material for: Nomogram for predicting cognitive impairment in middle-aged and elderly individuals with self-reported hearing loss: Insights from the longitudinal CHARLS cohort
Source: Braz J Otorhinolaryngol. 2026 Jan 7;92(2):101751. doi: 10.1016/j.bjorl.2025.101751 (PMC12813599; doi:10.1016/j.bjorl.2025.101751)
Supplement: Supplementary file 1 [file mmc1.docx]

**BJORL-D-25-00184**_ **Supplementary Material**

**Supplementary Table 1**

| **Variable Name** | **Definition** |
| --- | --- |
| Social Activity 1 | Visiting Friends or Socializing |
| Social Activity 2 | Leisure Activities (e.g., Mahjong, Chess, Cards) |
| Social Activity 3 | Providing Informal Support to Others |
| Social Activity 4 | Outdoor Physical Activities (e.g., Dancing, Exercise) |
| Social Activity 5 | Participation in Community Groups |
| Social Activity 6 | Volunteering or Charity Work |
| Social Activity 7 | Attending Educational or Training Programs |
| Social Activity 8 | Other Social Activities |
